# The Clinical Spectrum and Disease Course of DRAM2 Retinopathy

**DOI:** 10.3390/ijms23137398

**Published:** 2022-07-02

**Authors:** Tjaša Krašovec, Marija Volk, Maja Šuštar Habjan, Marko Hawlina, Nataša Vidović Valentinčič, Ana Fakin

**Affiliations:** 1 Eye Hospital, University Medical Centre Ljubljana, Grablovičeva ulica 46, 1000 Ljubljana, Slovenia; krasovec.tjasa@gmail.com (T.K.); sustar.majchi@gmail.com (M.Š.H.); marko.hawlina@gmail.com (M.H.); natasa.vidovic@kclj.si (N.V.V.); 2 Clinical Institute of Medical Genetics, University Medical Centre Ljubljana, Šlajmajerjeva ulica 4, 1000 Ljubljana, Slovenia; marija.volk@kclj.si; 3 Faculty of Medicine, University of Ljubljana, Vrazov trg 2, 1000 Ljubljana, Slovenia

**Keywords:** *DRAM2*, inherited retinal dystrophy, genetic spectrum, phenotype variability, genotype–phenotype correlation, fundus autofluorescence imaging, electrophysiology

## Abstract

Pathogenic variants in DNA-damage regulated autophagy modulator 2 gene (*DRAM2*) cause a rare autosomal recessive retinal dystrophy and its disease course is not well understood. We present two Slovenian patients harboring a novel *DRAM2* variant and a detailed review of all 23 other patients described to date. Whole exome and whole genome sequencing were performed in the two patients, and both underwent ophthalmological examination with a 2-year follow-up. PubMed was searched for papers with clinical descriptions of DRAM2 retinopathy. Patient 1 was homozygous for a novel variant, p.Met1?, and presented with the acute onset of photopsia and retina-wide retinopathy at the age of 35 years. The patient was first thought to have an autoimmune retinopathy and was treated with mycophenolate mofetil, which provided some symptomatic relief. Patient 2 was compound heterozygous for p.Met1? and p.Leu246Pro and presented with late-onset maculopathy at the age of 59 years. On review, patients with DRAM2 retinopathy usually present in the third decade with central visual loss, outer retinal layer loss on optical coherence tomography and a hyperautofluorescent ring on fundus autofluorescence. Either cone–rod or rod–cone dystrophy phenotype is observed on electroretinography, reflecting the importance of DRAM2 in both photoreceptor types. Non-null variants can result in milder disease.

## 1. Introduction

Inherited retinal dystrophies (IRD) are a group of clinically and genetically heterogenous conditions that are characterized by a progressive degeneration of the photoreceptors and the retinal pigment epithelium (RPE) cells [1,2,3,4]. Pathogenic variants in >250 genes are responsible for these diseases [5]. Among those, DRAM2 retinopathy is a rare, relatively recently discovered autosomal recessive IRD associated with pathogenic variants in *DRAM2*. *DRAM2* (MIM 613360) encodes a DNA-damage regulated autophagy modulator 2 (DRAM2), also known as transmembrane protein 77 (TMEM77), a 266 amino acid transmembrane protein containing six putative transmembrane domains [6,7]. It is ubiquitously expressed in numerous tissues, including the lymph nodes, spleen, heart, and placenta [8,9], where it localizes to lysosomal membranes and is thought to play a role in the autophagy process and tumor suppression [6,7,10,11]. Immunohistochemical analysis of the retina revealed that DRAM2 localizes to the inner segments of the photoreceptors and the apical surface of the RPE cells [12]. Since the first description of DRAM2 retinopathy in 2015 [12], fewer than 30 patients [12,13,14,15,16,17] have been reported with DRAM2 retinopathy, and the disease is still not well understood [12,13,14,15,16]. 

In the following paper, we present two Slovenian patients with a novel pathogenic variant in *DRAM2* and a detailed review of genetical and clinical characteristics of all DRAM2 cases described so far. The phenotypic spectrum is expanded with a late-onset presentation, and possible genotype–phenotype correlations are discussed.

## 2. Results

### 2.1. Characteristics of Slovenian Patients with DRAM2 Retinopathy

The results of genetic testing and clinical characteristics of Slovenian patients with *DRAM2* variants are summarized in Table 1. The variants were classified according to ACMG/AMP standards and guidelines for the interpretation of sequence variants [18].

#### 2.1.1. Genetic Findings

Genetic testing confirmed biallelic variants in *DRAM2* in both patients (Table 1 and Table 2). Patient 1 was homozygous for NM_001349881.1: c.3G > A (p.Met1?), while patient 2 was compound heterozygous for c.3G > A (p.Met1?) and NM_001349881.1:c.737T > C (p.Leu246Pro). The variant c.3G > A (p.Met1?) presented in both patients; it is novel and is predicted to cause the loss of the start codon in *DRAM2*. The variant is not reported in the gnomAD (gnomad.broadinstitute.org, accessed on 31 May 2022) or dbSNP (www.ncbi.nlm.nih.gov/snp/, accessed on 31 May 2022) databases. According to the ACMG/AMP criteria [18], it was classified as a variant with uncertain significance with the following grades: PVS1_MOD, and PM2. The missense variant c.737T > C (p.Leu246Pro) has been reported previously [17] and was classified as a variant with uncertain significance (PM2, PP3, PM3_MOD) according to the ACMG/AMP criteria [18]. 

#### 2.1.2. Clinical Findings

**Figure 1:**
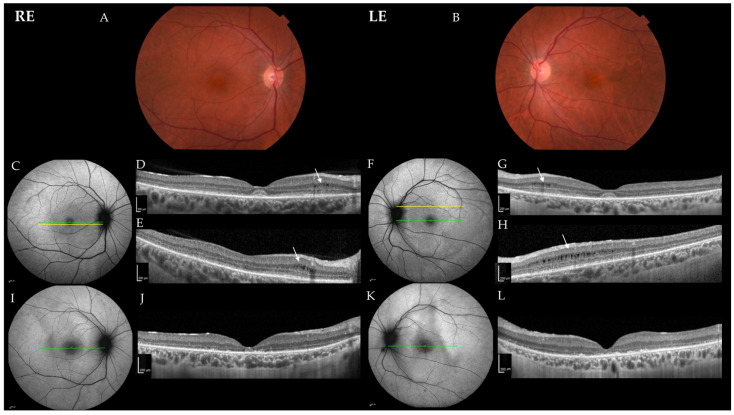
Color fundus photos and progression of DRAM2 retinopathy in Patient 1. (**A**,**B**) Color fundus photos at the age of 35 years; (**C**,**F**) fundus autofluorescence imaging (FAF) and (**D**,**G**) optical coherence tomography (OCT) images at the age of 35 years; (**E**,**H**) more peripheral OCT images at the age of 35 years; (**I**,**K**) FAF and (**J**,**L**) OCT images at the age of 37 years. Green lines in the FAF images show the location of the corresponding OCT scans in images (**D**,**G**,**J**,**L**). Yellow lines on the FAF images show the location of the corresponding OCT scans in images (**E**,**H**). Note the intraretinal cysts in images (**D**,**E**,**G**,**H**) (white arrows). On follow-up, the disappearance of the foveal photoreceptors (**J**,**L**) on and widening of the hypoautofluorescence in the fovea (**I**,**K**) is seen. Abbreviations: RE—right eye; LE—left eye.

**Figure 2:**
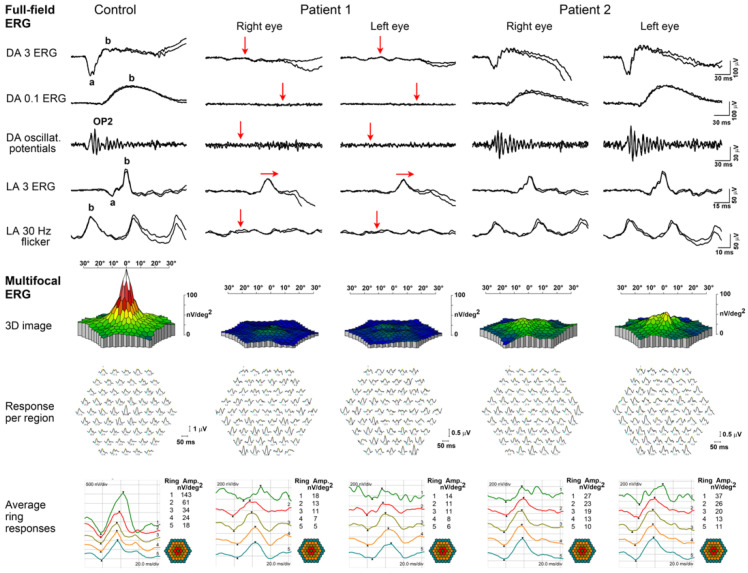
Full-field ERG and multifocal ERG findings in Patient 1 (last visit) and Patient 2 (first and only visit) compared with an age-matched control subject. Abbreviations: DA—dark-adapted; LA—light-adapted; Amp—amplitude; a—a-wave, negative ERG component that arises mostly from photoreceptoral activity; b—b-wave, a positive ERG component originating mostly from bipolar cells; OP2—oscillatory potential, second wave. Arrows indicate reduced (vertical arrow) and delayed (horizontal arrow) full-field ERG responses in Patient 1. Note the reduced mfERG responses in Patient 1 over the whole macular region. The mfERG responses in Patient 2 were reduced, especially in the inner two rings.

**Figure 3:**
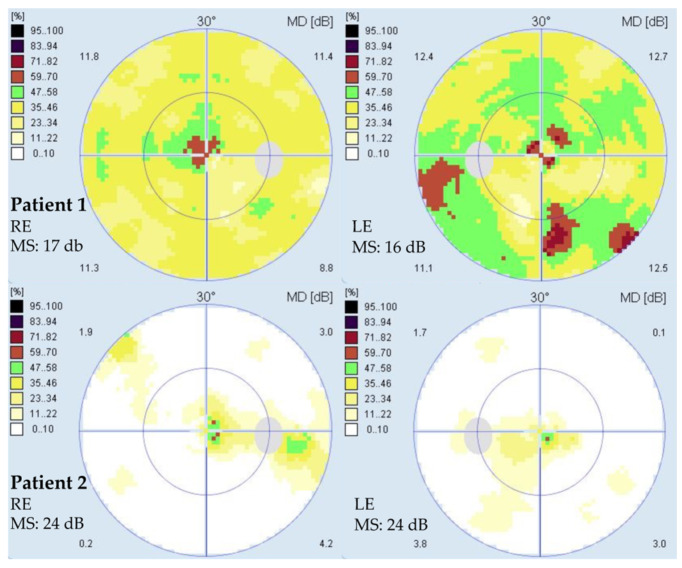
Visual field (Octopus G2 top) of Patient 1 (**upper row**) and Patient 2 (**bottom row**) at the last visit at the ages 37 and 63 years, respectively. Note the reduced central sensitivity in both patients, more pronounced in Patient 1. A color-coded sensitivity scale is shown on the left. Abbreviations: RE—right eye; LE—left eye; MS—mean sensitivity.

The clinical findings are summarized in Table 1. Patient 1 presented at the age of 35 years with a one-month history of quickly worsening pericentral visual loss and photopsia in both eyes (BE). At first exam, her best corrected visual acuity (BCVA) was 1.0 in BE, and she had normal color vision in BE (Ishihara 14/15). A slit lamp exam revealed 1+ to 2+ cells in the vitreous fluid, an absent foveal reflex granular macular appearance and parafoveal yellow dots in BE. Fundus autofluorescence imaging (FAF) showed poorly delineated hypoautofluorescent and hyperautofluorescent lesions in the macula, and optical coherence tomography (OCT) showed a severe loss of the photoreceptor layers in the parafoveal region in BE, with relative foveal sparing and intraretinal cysts in the regions of still preserved photoreceptors (Figure 1). She had bilaterally reduced macular sensitivity on static perimetry with a mean sensitivity (MS) in the right eye (RE) of 25 dB and in the left eye (LE) of 22 dB. Electroretinography (ERG) findings were as follows: pattern ERG (PERG) revealed macular dysfunction, and multifocal ERG (mfERG) localized the dysfunction to the foveal and parafoveal regions (mfERG responses were reduced in the inner two rings) in BE. Full-field ERG (ffERG) showed the generalized impairment of retinal function with reduced rod- and delayed cone-specific responses. An extensive workup was performed due to the acute onset. Neoplastic process was excluded with head magnetic resonance imaging (MRI), chest X-ray and positron emission tomography (PET-CT), and paraneoplastic panel was negative except for borderline positive Zinc finger protein (ZIC4) antibodies. Uveitis screening was also negative, except for a low antinuclear antibody (ANA) titer (1:320 on immunofluorescence assay). Due to rapid worsening, a diagnosis of autoimmune retinopathy was presumed, and the patient received six pulses of methylprednisolone 500 mg and eight cycles of plasmapheresis. Methylprednisolone was tapered off with prednisolone, and she continued with Mycophenolate Mofetil (MMF) 1000 mg 2× daily. After being diagnosed, the patient was taking cannabidiol on her own as she self investigated that it affected autophagy, but we do not know the dose and duration of this treatment. Over 26 months of follow-up, she showed slow continuous worsening of BCVA, with worsening of visual field on static perimetry, structural deterioration on OCT, additional reduction of mfERG responses over the whole macular region, undetectable rod-specific response on the ffERG and severely delayed cone-specific response (Figure 2). The patient reported subjective worsening when discontinuing treatment with MMF. At the last visit after 26 months of follow-up, her BCVA was 0.6 in RE and 0.5 in LE, color vision was reduced (Ishihara 5/11 in RE and 3/11 in LE) and there was worsening of mean sensitivity on static perimetry (MS in RE 17 dB and in LE 16 dB) (Figure 3); on kinetic perimetry (Campus Goldmann), she did not see isopter II/1, and isopters II/2 and II/3 were narrowed in BE. Slit lamp examination revealed 1+ to 2+ cells in the vitreous fluid. OCT showed a loss of the photoreceptors in the fovea in BE (in the inner segment ellipsoid (ISe) band), and FAF revealed more widespread hypoautofluorescent and hyperautofluorescent lesions towards the periphery in BE (Figure 1).

**Figure 4:**
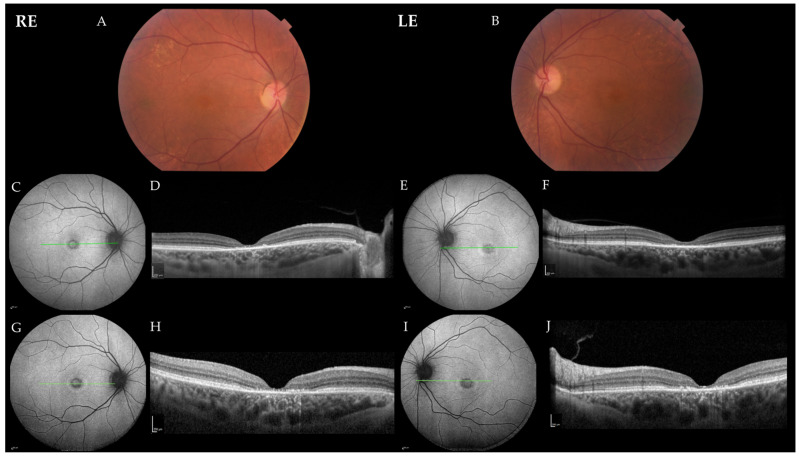
Color fundus photos and progression of DRAM2 retinopathy in Patient 2. (**A**,**B**) Color fundus photos at the age of 63 years; (**C**,**E**) fundus autofluorescence (FAF) and (**D**,**F**) optical coherence tomography (OCT) images at the age of 61 years; (**G**,**H**) fundus autofluorescence and (**I**,**J**) and OCT images at the age of 63 years. Green lines in the FAF images show the location of the corresponding OCT scans. Note a minimal darkening of the foveal hypoautofluorescence corresponding to the increased signal passing through the choroid on the OCT, reflecting retinal pigment epithelium atrophy. Abbreviations: RE—right eye; LE—left eye.

Patient 2 presented at the age of 61 years with a two-year history of the slow bilateral deterioration of vision with reading difficulties, problems recognizing faces and photophobia. At exam, her BCVA was 0.2 in BE, and she had normal color vision (Ishihara 14/15 in RE and 15/15 in LE). She had central scotoma on static perimetry (MS in RE 27 dB and in LE 26 dB) and slightly narrowed visual field on kinetic perimetry (Campus Goldmann). Slit lamp exam revealed nuclear cataract in BE, whereas fundus examination revealed an absent foveal reflex, narrow blood vessels, the granular appearance of the macula and yellow dots around the vascular arcades in BE (Figure 4). OCT showed absent photoreceptor layer in the fovea in BE, and FAF showed foveal hypoautofluorescence delineated by a hyperautofluorescent ring in BE (Figure 4). ERG findings were consistent with macular dysfunction; mfERG showed reduced responses, especially in the inner two rings in BE, whereas ffERG was still within the normal range (Figure 2). After 25 months of follow-up, there was no significant deterioration of visual function, her BCVA was 0.2 in BE, color vision was still preserved (13/15 in RE and 14/15 in LE) and the fundus was similar to the first exam. OCT and FAF showed minimal progression of the disease (Figure 4), and visual field showed reduced MS (BE 24 dB) (Figure 3).

### 2.2. Review of Previously Published Cases with Variant in DRAM2

The search in the previously published cases of retinopathy caused by biallelic variants in *DRAM2* identified 6 reports with overall 24 cases from 14 families [12,13,14,15,16,17]. The genetic and clinical findings of these cases including ours are summarized in Table 2 and Table 3, respectively. 

#### 2.2.1. Genetic Findings

Including this report, 19 unique variants in *DRAM2* associated with retinopathy caused were identified (Table 2). Among these, 42% (8/19) are missense, 26% (5/19) in-frame deletion/insertion, 16% (3/19) nonsense, 11% (2/19) splicing and 5% (1/19) start loss. Of the variants, 42% (8/19) were considered a variant of unknown significance, 47% (9/19) variants were likely pathogenic and two of them (11%; 2/19) were considered pathogenic. 16% (3/19) variants were considered to be null (Table 2). Variants were reported in either homozygous state (58%; 11/19) or in compound heterozygous state (42%; 8/19). In the patients, 81% (21/26) were homozygotes and 19% (5/26) were compound heterozygotes (Table 2).

#### 2.2.2. Clinical Characteristics

**Figure 5:**
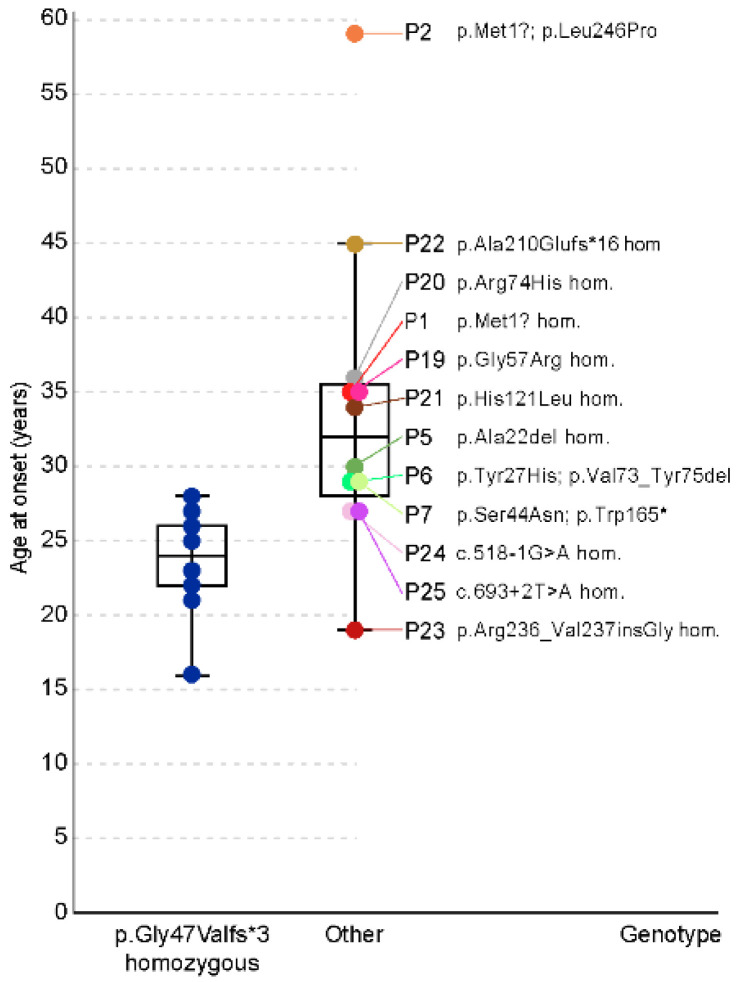
Disease onset in patients harboring homozygous variant p.Gly47Valfs*3 (baseline group) compared with the other patients. Note the significantly earlier median age of onset in the baseline group. Other patients had very variable disease onsets i.e., Patient 2 (p.Met1?; p.Leu246Pro) at the age of 59 years, Patient 22 (p.Ala210Gluufs*16 homozygous) at the age of 45 years and Patient 23 (p.Arg236_Val237insGly homozygous) at the age of 19 years. Note that all patients with pathogenic variant p.Gly47Valfs*3 are in the blue color. The colors of the patients are the same in Figure 5, Figure 6, Figure 7 and Figure 8.

The clinical characteristics of all DRAM2 patients are summarized in Table 1 (Slovenian patients, N = 2) and Table 3 (other patients, N = 23). Patient 26 had IRD, but his detailed clinical description is not available. Among the patients with available data (N = 24), 38% (9/24) were male and 62% (15/24) were female. The median age of the onset of initial visual symptoms was 27 (range 16–59) years (Figure 5). All patients were symptomatic, except one who was diagnosed at the age of 19 years in genetic testing performed due to family history. The most frequent symptom at onset among the symptomatic patients was central visual loss (63%; 15/24). Other reported symptoms included progressive visual loss with photophobia (17%; 4/24), nyctalopia (8%; 2/24), pericentral visual loss (4%; 1/24), reduction of visual acuity (4 %; 1/24) and dark adaption difficulties (4%; 1/24).

**Figure 6:**
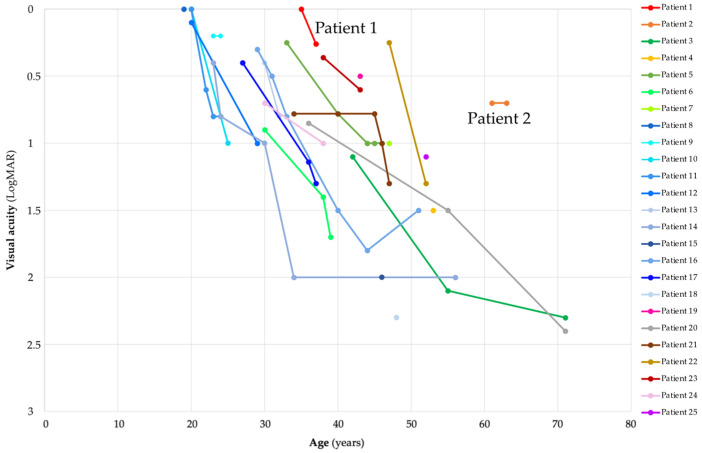
Visual acuity in patients with DRAM2 retinopathy and its worsening through follow-up. Patient 1 (p.Met1?; p.Met1?) had relatively good visual acuity in comparison with other patients harboring two start loss. Patient 2, harboring one missense allele (p.Met1?; p.Leu246Pro), had noticeably better visual acuity in comparison with the other patients. Note that all patients with pathogenic variant p.Gly47Valfs*3 are in the shades of blue. Note that the colors of the patients are the same in Figure 5, Figure 6, Figure 7 and Figure 8.

The median BCVA at first exam (N = 25) (median age 34; range 19–61 years) in the better eye was 0.4 Snellen decimal (range 0.005–1.0) or 0.4 (range 0–2.3) LogMAR (Figure 6). The median BCVA at the last exam (N = 18) (median age 41; range 24–63 years) was 0.1 Snellen decimal (range 0.005–0.6) or 1.0 (range 0.2–2.3) LogMAR (Figure 6). 

#### 2.2.3. Slit-Lamp Findings

The fundus appearance description at the first exam (median age 38; range 19–61 years) most often included macular atrophy (68%; 17/25) or macular degeneration (16%; 4/25), accompanied by granular macular appearance (64%; 16/25) and/or with white/yellow dots (48%; 12/25). Some patients had isolated fundus findings, such as yellow dots in the macula (Patient 3), granular macular appearance (Patient 4) or irregular foveal reflex (Patient 9). The asymptomatic Patient 8 presented without abnormal findings. Changes towards the periphery were observed at a median age of 50 (32–71) years. Among other findings, 32% (8/25) of the patients had pigmentary depositions (bone–spicule degeneration and/or widespread pigment clumping) at first exam (median age 50; range 32–61 years), and 44% (11/25) of patients had follow-up data after the median of 7 (range 2–29) years. Pigmentary depositions were noted in 82% (9/11) patients with follow-up data. Except for Patient 1, no patients had cells noted in the vitreous fluid.

#### 2.2.4. OCT Characteristics

OCT data were available for 20 patients, 19 symptomatic and 1 asymptomatic. Outer retinal layer loss was observed in most of the symptomatic patients (84%; 16/19). One symptomatic patient (Patient 10) presented with reduced foveal thickness at the age of 21, which progressed to outer retinal layer loss at the age of 25. Foveal thinning due to severe atrophy of the photoreceptor and RPE layers was observed in Patient 22, and disrupted ellipsoid zone with thinning of the outer retinal layers at the macula was seen in Patient 23. OCT images of the asymptomatic individual (Patient 8) showed reduced foveal thickness with perifoveal disruption of the ellipsoid zone. Among the patients with available OCT images (N = 11), 18% (2/11; Patient 1 and Patient 4) had preserved integrity of the Ise band and/or external limiting membrane. In Patient 1, the integrity of ISe was disrupted after 2 years.

#### 2.2.5. FAF Characteristics

**Figure 7:**
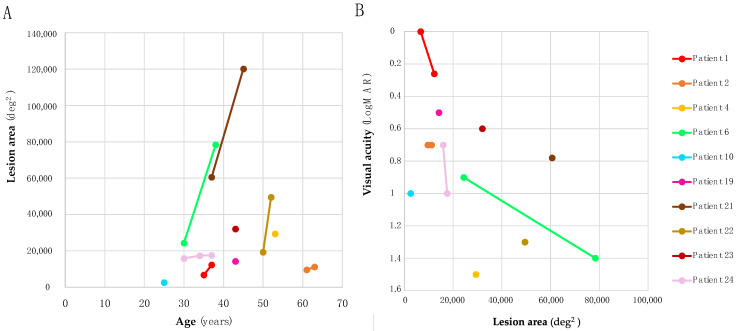
(**A**) The correlations between age and lesion area on fundus autofluorescence imaging. All patients with longitudinal imaging showed increases in lesion area with time. Note the relatively small lesion area size of Patient 2, harboring p.Met1? and p.Leu246Pro. (**B**) The correlations between lesion area and visual acuity on fundus autofluorescence imaging. Note the relatively large lesion area size and good visual acuity of Patient 21 at the age of 37 years. Visual acuity in this patient deteriorated to 1.3 LogMAR after 10 years, but his OCT images for evaluating the integrity of his photoreceptor layer are not available. On the other hand, Patient 4 had relatively small lesion area size and poor visual acuity (1.5 LogMAR). On OCT, the integrity of external limiting membrane was still preserved in this patient. Note that the colors of the patients are the same in Figure 5, Figure 6, Figure 7 and Figure 8.

FAF data were available for 14/25 patients, all of whom were symptomatic. The most frequent pattern observed on FAF was a central hypoautofluorescent area surrounded by a hyperautofluorescent ring (57%; 8/14). Patient 4 had a paracentral hyperautofluorescent ring and central thinning of the photoreceptor layer. Perifoveal hypoautofluorescence was seen in Patient 22 and Patient 24, which in both progressed to more diffuse through follow-up. Patient 25, who was older than 22 and 24, had greater retinal hypoautofluorescence that was seen in the macula and periphery. FAF images from Patient 1 and Patient 20 showed a mosaic pattern with hyperautofluorescence and hypoautofluorescence. Of the patients, 36% (9/25) had follow-up data after the median of 4 (range 2–8) years. FAF images from follow-up visits revealed progression of the disease (enlargement of the central defect towards midperiphery and periphery) in six individuals (67%; 6/9) with median age of 55 (range 38–54) years. In patients with available FAF images (N = 10), we measured the area of definite decreased autofluorescence. The correlation between age and lesion area on FAF (at the last exam) was not statistically significant (Pearson correlation, *p* = 0.97) (Figure 7A), and the correlation between lesion area on FAF and visual acuity was also not statistically significant (Pearson correlation, *p* = 0.15) (Figure 7B).

#### 2.2.6. ERG Characteristics

The results for PERG (5/25), mfERG (5/25), ffERG (14/25) and flicker ERG (4/25) were inspected for all patients. On PERG, all patients (100%; 5/5) presented with abnormal responses indicating severe macular dysfunction. Similarly, mfERG indicated macular dysfunction in all patients (100%; 5/5). In a group that included Patient 2, 43% (6/14) of patients initially presented with normal ffERG responses (median 39; range 30–61 years), which in Patient 6 and Patient 21 deteriorated to subnormal under both dark- and light-adapted conditions. Another 21% (3/14; among them also Patient 1) had reduced to almost nonrecordable both rod- and cone-specific responses, whereas 21% (3/14) had reduced cone-specific and almost undetectable (Patient 3) to nondetectable (Patient 20) rod-specific responses. Undetectable ffERG was recorded in two patients (Patient 7 and Patient 25; 14%, 2/14), who were also among the oldest (47 and 52 years, respectively). Flicker ERG was severely attenuated and connected with macular dysfunction in three patients (75 %; 3/4), whereas it did not show delay in one patient with macular atrophy (Patient 19; 25 %, 1/4). Based on ERG, 29% (4/25) of patients had macular dysfunction, 50% (7/14) had a pattern of cone-rod dystrophy (CRD) and 21% (3/14) had a pattern of with rod-cone dystrophy (RCD) with macular involvement.

#### 2.2.7. Genotype-Phenotype Correlations

**Figure 8:**
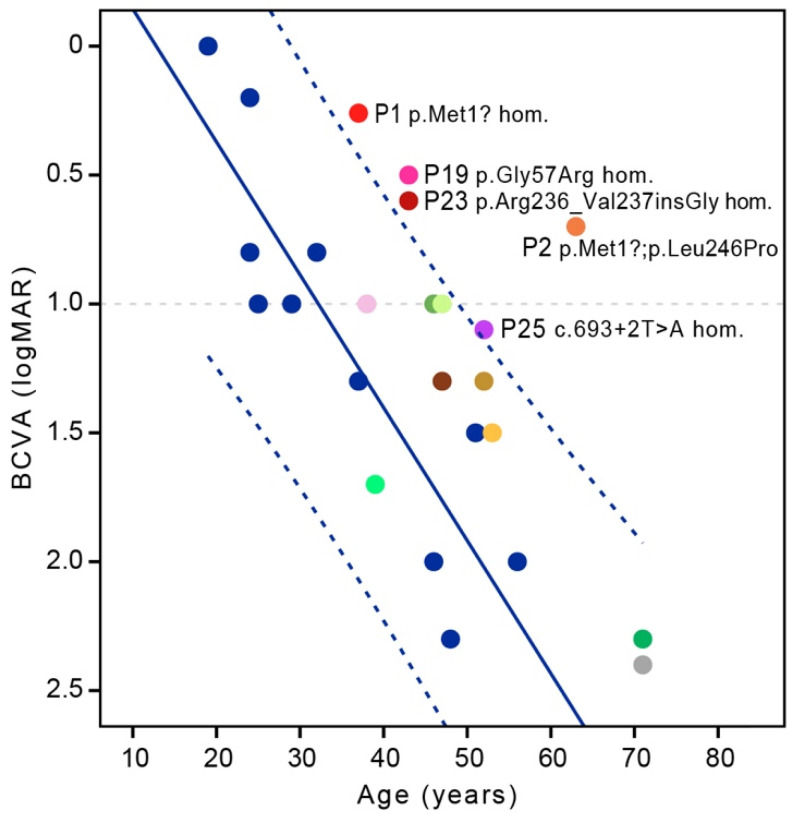
Visual acuity at last exam in patients harboring variant p.Gly47Valfs*3 (blue color) and other biallelic variants (other colors, all homozygotes are in the shades of blue) in *DRAM2*. Five patients had visual acuity above the 95% CI of the baseline group: Patient 1 (p.Met1? homozygous), Patient 2 (p.Met1?; p.Leu246Pro), Patient 19 (p.Gly57Arg homozygous), Patient 23 (p.Arg236_Val237insGly homozygous) and Patient 25 (c.693 + 2T > A homozygous). Note that all patients with pathogenic variant p.Gly47Valfs*3 are in the blue color. Note that the colors of the patients are the same in Figure 5, Figure 6, Figure 7 and Figure 8.

We aimed to determine if any genotypes result in a notably milder phenotype. The most frequent genotype was Gly47Valfs*3 in homozygous state, present in 11 patients (Patients 8–18) from the same family of Pakistani origin [12]. The patients with this genotype (baseline group) had relatively more severe course of disease in comparison and other patients (others) (Figure 5, Figure 6 and Figure 8). The genotype–phenotype correlation was statistically significant for several parameters. 

Disease onset was significantly earlier in the baseline group (median 24; range 16–28 years) than in others (median 32; range 19–59 years) (Mann–Whitney test, *p* < 0.05) (Figure 5). Patient 23 (p.Arg236_Val237insGly homozygous) had earlier disease onset (19 years) than the median age of the baseline group. Four patients had onset of the disease in the 4th decade: Patient 5 (p.Ala22del homozygous; 30 years), Patient 19 (p.Gly57Arg homozygous; 35 years), Patient 20 (p.Arg74His homozygous; 36 years) and Patient 21 (p.His121Leu homozygous; 34 years) (Figure 5). One patient had onset of the disease in the 5th decade (Patient 22; p.Ala210GlufsTer16 homozygous; 45 years) and one patient in the 6th decade (Patient 2; p.Met1?; p.Leu246Pro; 59 years) (Figure 5). BCVA at last exam for patients homozygous for variant Gly47Valfs*3 was worse than for other patients when corrected for age (multiple regression, *p* < 0.01) (Figure 8). Accounting for visual acuity decline with age, five patients had BCVA above the 95% confidence interval (CI) of the baseline group (Figure 8). They were Patient 1 (p.Met1? homozygous), Patient 2 (p.Met1?; p.Leu246Pro), Patient 19 (p.Gly57Arg homozygous), Patient 23 (p.Arg236_Val237insGly homozygous) and Patient 25 (c.693 + 2T > A homozygous). Patient 22, homozygous for the truncating variant p.Ala210GlufsTer16, had relatively good BCVA (Figure 6 and Figure 8) but was at the last exam within the 95% CI of the baseline group (Figure 8).

## 3. Discussion

This study provides a comprehensive overview of the disease spectrum of DRAM2 retinopathy. We extend the cohort of 16 patients described by El-Asrag et al. [12] and Sergouniotis et al. [13] with an additional 10 patients, including two Slovenian patients with a novel variant, one with an acute onset of widespread retinopathy mimicking autoimmune retinopathy and the other with a mild, late-onset maculopathy. Additionally, we performed an analysis of all reported FAF images and propose genotype–phenotype correlations. 

### 3.1. Disease Onset 

Based on the review all (N = 25) DRAM2 patients with clinical data, the first symptoms most often appear in the third decade [12,13,14,15,16], although the onset varies from teenage years to the sixth decade of life (Figure 5). The earliest reported onset was by Patient 18 at the age of 16 [12], whereas the Slovenian Patient 2 had the latest onset so far, at the age of 59 years (Figure 5). This finding expands the phenotypic spectrum of DRAM2 retinopathy with a late-onset presentation, as previously the latest reported onset was at the age 45 years [15]. If the disease occurs late in life and is mild, as in Patient 2, in whom the peripheral retina was spared, it may be confused with age-related macular degeneration. It is therefore possible that late-onset patients are undiagnosed and that the prevalence of DRAM2 retinopathy is higher than currently thought. A large range in disease onset has also been observed in other retinal dystrophies, including the most frequent monogenic disease, ABCA4 retinopathy [19]. There, the variability in onset is to some extent linked to the type of genetic defect [20], and it is likely that the same is true for DRAM2 retinopathy. Slovenian Patient 2 with the latest disease onset harbored a combination of a start loss (p.Met1?) and a missense variant (p.Leu246Pro). The other six patients with relatively late disease onset (30–39 years) also harbored presumably non-null variants, namely p.Met1?; p.Leu246Pro (Patient 2), p.Ala22del homozygous (Patient 5), p.Gly57Arg homozygous (Patient 19), p.Arg74His homozygous (Patient 20), p.His121Leu homozygous (Patient 21) and p.Ala210GlufsTer16 homozygous (Patient 22). We presume that certain alleles, such as p.Leu246Pro, retain some residual function of DRAM2 protein; however, a larger cohort of patients with the same genotypes and/or in vitro studies are needed to confirm this. The first symptom in DRAM2 retinopathy is usually central visual loss, which was reported by 63% (15/24) patients and is consistent with early macular impairment [12,13,21]. In addition, some patients (17%; 4/24) reported photophobia. This is a relatively frequent symptom in patients with IRDs and is thought to reflect an early involvement of photopigment-containing cells [15,22]. It is suggested that light hypersensitivity occurs due to impairment of either S-cones in the parafoveal region, rods, and/or photosensitive retinal ganglion cells, but the mechanism is not fully understood [22]. Interestingly, a subset of DRAM2 patients (12%; 3/24) described difficulty seeing in dark environments and dark adaption difficulties as their initial symptoms, suggesting an early rod impairment [14,16]. Their genotypes were p.Trp3del homozygous (Patient 3) and p.Val16Ala; p.Gly95Val (Patient 4). Although nyctalopia is often observed in patients with CRD, it is not expected to be the first symptom [16,23]. Patient 4 had reduced dark-adapted and light-adapted responses, whereas Patient 3 had ERG findings in the pattern of RCD.

### 3.2. Visual Acuity

The median BCVA at first exam (median age 34; range 19–61 years) in the better eye was 0.4 Snellen decimal, deteriorating to 0.1 at last exam (median age 41; range 24–63 years) (Figure 6). This reflects the progressive nature of DRAM2 retinopathy, with mostly poor visual outcomes after the fifth decade. The best preserved BCVA was noted in Slovenian Patient 2, who at the age of 63 years still had BCVA of 0.2 Snellen decimal (0.7 LogMAR) (Figure 6). Several other patients also had notably better preserved BCVA for their age in comparison with patients homozygous for p.Gly47Valfs*3 (baseline group) (Figure 8).

### 3.3. Fundus Appearance

Fundus examination in both Slovenian patients and previously reported cases [12,13,14,15,16] reveals that degeneration initiates in the macula (Figure 1 and Figure 4). First signs were in most cases described as macular atrophy and/or granular macular appearance (Table 1 and Table 3). Tiny white or yellow dots were observed in 48% (12/25) of patients, including in the Slovenian patients. Considering the DRAM2 role in autophagy, the dots could represent the residual components of the visual cycle [11]. Although probably not pathognomonic, they could potentially help differentiate DRAM2 retinopathy from other dystrophies. At first exam, 32% (8/25; median age 50; range 32–61 years) patients had pigmentary depositions (bone–spicule degeneration and/or widespread pigment clumping), and it was noted in most patients with follow-up exams (82%; 9/11), suggesting progressive peripheral degeneration [15,16]. According to the fundus appearance, disease progression is rather slow, with changes towards periphery observed at a median age of 50 (range 32–71) years. Only one (8%; 1/12) patient older than 45 years had no peripheral changes (Patient 4).

### 3.4. OCT

Outer retinal loss was observed on OCT in most (84%; 16/19) of the symptomatic patients as well as the one asymptomatic patient. This is consistent with the finding that DRAM2 localizes to the outer retina, i.e., the photoreceptor layer and the apical surface of the RPE [12]. The absence of DRAM2 in the retina is thought to reduce the effectiveness of autophagy, leading to diminished photoreceptor renewal [12]. In the asymptomatic individual (Patient 8), OCT images revealed perifoveal disruption of the ellipsoid zone, which suggests that the initial impairment began in the photoreceptor layer [12]. On the other hand, considering that autophagy in RPE is important for the degradation of the outer segments of photoreceptors, the degeneration could also begin in the RPE [13]. Interestingly, in Patient 1, the loss of photoreceptor layers also began in the parafoveal region with relative foveal sparing, which coincided with her good BCVA at the first exam. However, after 2 years, her vision deteriorated, and the loss of photoreceptor in the foveal and perifoveal regions was observed. In total, 18% (2/11) of patients with available OCT images had preserved photoreceptors in the fovea when parafoveal degeneration had already occurred. It is possible that patients who had loss of central vision and outer retinal layer loss on OCT at the first presentation also had perifoveal impairment a few years before and only reported to the ophthalmologist when degeneration spread to the fovea. Kuniyoshi et al. [16] showed pericentral scotoma in two patients that expanded to involve the whole macula in the course of disease (Patient 3 and Patient 20 in this review). The pattern of initial foveal sparing and/or initial perifoveal degeneration is not specific for DRAM2 retinopathy and has been noted in several other monogenic and multifactorial diseases. These include macular diseases such as age-related macular dystrophy and cone-rod dystrophies such as foveal sparing ABCA4 retinopathy and certain PRPH2 retinopathies [24,25,26]. RCD may also begin with an annular scotoma and in late stage also often exhibits perifoveal RPE atrophy, surrounding the preserved RPE in the fovea [27]. These patterns of degeneration are thought to be influenced by disease independent factors such as metabolic differences between regions of the macula, rod-derived cone viability factor, variations in macular pigment and peak distribution, cone density, increased vulnerability in certain parafoveal photoreceptors, factors related to RPE and choroid, etc. [28].

### 3.5. FAF

The most frequent FAF pattern in DRAM2 patients was central hypoautofluorescent area surrounded by a hyperautofluorescent ring, present in 57% (8/14) of patients, corresponding with the area of photoreceptor loss on OCT (Figure 4). The hyperautofluorescent ring may be found in several IRDs and usually delineates the border between the preserved and affected retina. In cone dystrophy (CD)/CRD, the retina is affected inside the ring, whereas in RCD (retinitis pigmentosa (RP)), the retina is affected outside the ring [29,30,31]. The source of hyperautofluorescence is thought to be photoreceptor outer segment loss overlaying the still intact RPE, resulting in the increased detection of the normal RPE autofluorescence due to the decreased blockage of photoreceptors [13]. Another cause of increased autofluorescence is possibly the increased accumulation of lipofuscin in the degenerating photoreceptor inner segments and/or the RPE, potentially contributing to photoreceptor damage [29,30,32].

The hypoautofluorescence within the ring in DRAM2 corresponded to the RPE atrophy, which presumably followed photoreceptor loss, as has been observed in other CRD patients [30]. FAF images from follow-up visits showed the enlargement of the hyperautofluorescent rings, a clinical feature that suggests disease progression over time and that has also been reported in patients with other CRD [31,33,34,35]. The enlarging of central hypoautofluorescence is consistent with expanding macular atrophy, and the expansion of the hyperautofluorescent ring probably reflects the disorganization of the photoreceptors during the progress of the disease [31,33]. Slovenian Patient 1 did not exhibit a clear hyperautofluorescent ring but instead, larger hyperautofluorescent lesions in the macula, corresponding with photoreceptor loss on OCT (Figure 1). From the available images for published cases (N = 14), Patient 20, who is also the oldest patient (71 years old; p.Arg74His homozygous), exhibited this finding. Furthermore, 43% (6/14) of patients with at least 55° imaging showed hypoautofluorescent lesions along the midperiphery, representing widespread retinal disease. Interestingly, their median age (44; range 38–54) was similar to the patients without midperipheral lesions (45; range 25–71). As in other retinal dystrophies, FAF and OCT are useful imaging tools for evaluating the structural damage of the retina and following the disease progression in patients with DRAM2 retinopathy. 

### 3.6. ERG

First reports of DRAM2 retinopathy described findings consistent with CRD [12,13,14,15,16]. Later, Kuniyoshi et al. [16] observed that some patients exhibit a RCD phenotype. Macular dystrophy was also reported [12,13]. On review, among patients with ERG data (N = 14), 50% had ERG features of CRD, 21% of RCD and 29% of macular dystrophy with normal full-field ERG. In CD/CRD, cones are primarily targeted, which results in central visual loss, a remarkable decrease in visual acuity and central scotoma [1]. In CRD, rod impairment and rod-related symptoms such as loss of peripheral vision and night blindness appear at subsequent stages [1,3]. On the other hand, RCD (RP) is characterized by primary rod degeneration followed by cone degeneration. These patients usually present with night blindness and progressive loss of the peripheral visual field that is followed by loss of central vision due to cone impairment [1]. It is important to note that ERG diagnosis of a RCD does not always align with the clinical diagnosis. For example, typical RCD (RP) is supported with structural findings of peripheral retinal involvement and relative sparing of the macular area. None of the DRAM2 patients were reported to exhibit this phenotype as they all exhibited early macular involvement except for initial foveal sparing in some cases. Patient 23 presented with difficultly in night vision at the age of 19 years, which could suggest primary rod impairment. However, she lost peripheral visual field only at 43 years, at the same time as her ffERG responses were undetectable. A couple of years before that, she already had a central scotoma, while ffERG responses were still in normal range. Considering clinical and ERG findings together, her presentation is more consistent with CRD. In keeping with that, among the two Slovenian patients, Patient 1 (homozygous for p.Met1) had a greater reduction of rod- than cone-specific function on ffERG (Figure 2) but with a predominant macular involvement. At presentation, the ERG findings were not typical of CRD but were more like retinopathy of inflammatory origin. Typically, CRD patients have severely abnormal mfERG and PERG that corelates with poor visual acuity [36], whereas she still had good visual acuity, although perifoveal structural loss was seen. In addition, ffERG in CRD typically shows preserved but reduced rod- and cone-specific responses [37], whereas the patient had reduced rod-specific responses while the responses of cone system were only delayed. This suggests that cones were structurally still largely preserved at that time but that their functioning was disturbed. Intraretinal cysts that were observed at that time in regions of preserved photoreceptors in the macula (Figure 1) are also in concordance with the existence of an inflammatory process. On the other hand, Patient 2 (p.Met1?; p.Leu246Pro) had abnormal mfERG and normal ffERG (Figure 2), a finding suggestive of macular dystrophy. The homozygous nonsense variant (p.Met1?) that Patient 1 carries seems to lead to CRD, whereas the same variant in the biallelic state (p.Met1?; p.Leu246Pro) that Patient 2 carries seems to lead to macular dystrophy. However, studies on a larger cohort of patients will be needed to determine whether there exists some genotype–phenotype correlation or if any genetic, epigenetic modifiers and environmental factors affect the phenotype. Considering all these observations, DRAM2 retinopathy most commonly leads to CRD, less often to atypical CRD with early rod dysfunction or macular dystrophy and never to typical RCD. A similar observation has been proposed in *EYS*, which mostly causes RP, i.e., RCD, but has also been described as a cause of CRD and macular dystrophy that further confirms the clinical heterogeneity of IRDs [38].

### 3.7. Genotype–Phenotype Correlations

We identified a novel variant c.3G > A (p.Met1?) in the *DRAM2*. The variant is predicted to cause the loss of the start codon with a novel start codon downstream. Both Slovenian patients carried this variant. A priori predictions of start loss variant effect on the final protein structure is difficult to determine. It has been shown that there are non-canonical or non-AUG translation initiation sites that are used by the cell to warrant protein translation. These sites are generally not as effective as the canonical ones, but they still guarantee a minimum production of the protein. In addition, different protein isoforms may have a different start codon and can make up the lack of one of the others. Therefore, functional in vitro studies are necessary to demonstrate the actual biological effect of start-loss variant [39,40,41]. Patient 1 was homozygous and presented with a widespread retinopathy, while Patient 2 was compound heterozygous for c.3G > A (p.Met1?) and a missense variant c.737T > C (p.Leu246Pro) and presented with a late-onset maculopathy. We presume that the disease in Patient 2 was milder and delayed mostly due to the residual function conferred by the missense variant. However, it is possible that p.Met1? also retains some DRAM2 function. Compared to the baseline group of patients with two null variants, the Patient 1, homozygous for p.Met1? had a delayed disease onset (35 years vs. median 24 years) and significantly better preserved visual acuity (Figure 8). Nevertheless, the disease was still affected the whole retina and progressed relatively quicky, thus the retained function, if present, is not enough to prevent a severe disease.

Interestingly, although the 26 patients (25 with clinical data) had 19 different variants, 81% (21/26) patients were homozygous. The major reason for this seems to be consanguinity, which was noted for Patients 8–18 from the same large family of Pakistani origin [12,13] as well as Patient 5 [12,13], Patients 22 and 25 [15], Patient 3 and Patient 23 [16]. The other possible reason is that different variants are founder variants in different geographic regions. Slovenian homozygous patient had no history of consanguinity but does come from a small geographic region. It is possible that the variant p.Met1? is a founder variant for the Slovenian region. A similar observation was made for Usher patients in Slovenia, where variant c.11864G > A (p.Trp3955*) in represent the majority (84%) of pathogenic alleles in Slovenian USH2A Usher syndrome population, and is otherwise a rare variant [42]. In the previous studies of DRAM2 retinopathy it was suggested that transcripts of variants in Patient 7 (p.Ser44Asn; p.Trp165*), Patients 8–18 (p.Gly47Valfs*3 homozygous), Patient 22 (p.Ala210GlufsTer16 homozygous) and Patient 24 (c.518-1G > A homozygous) create premature termination codons (PTC), which are eliminated by the nonsense-mediated mRNA decay (NMD) to avoid aberrant gene expression [12,13,15,43]. In addition to that, Abad-Morales et al. [15] observed that DRAM2 mRNA expression in Patient 22 and Patient 24 was decreased, coinciding with PTC and promoting NMD transcript degradation. They had similar phenotypes with mid-peripheral RPE disturbances. In Patient 25 no differences in DRAM2 mRNA levels were detected, because skipping exon 8 does not affect the open reading frame. Interestingly, the Patient 25 had more severe and widespread degeneration in this study and the author proposed it is due the requirement of exon 8 for the correct biological function of the protein [15]. Sergouniotis et al. [13] concluded that individuals harboring at least one loss-of-function variant present first symptoms earlier than patients harboring only missense variants or in-frame deletions. Similar observations were made in other retinal dystrophies. For example, in ABCA4 retinopathy, the BCVA and lesion area involvement were significantly more severe in the patients with two null variants than in patients harboring null and missense variant or two missense variants [44]. In our review, the patients with biallelic variant p.Gly47Valfs*3 (baseline group) BCVA at first exam had significantly worse phenotype in comparison to most patients with other biallelic variants (Figure 8). One option is that some stop variants escape the NMD and some splicing variants partially retain normal splicing. Considering that patients with p.Gly47Valfs*3 were from the same family, other non-DRAM factor could influence their phenotype. The genotypes that were consistently better than the baseline group were namely p.Met1? homozygous (Patient 1), p.Met1?; p.Leu246Pro (Patient 2) p.Gly57Arg homozygous (Patient 19) and p.Ala210GlufsTer16 homozygous (Patient 22). On the other hand, some genotypes were relatively similar to the baseline group, e.g., c.518-1G > A homozygous (Patient 24) and c.693 + 2T > A homozygous (Patient 25). The missense variant of Patient 7 p.Ser44Asn; p.Trp165*) also likely does not retain much DRAM2 function considering the patient’s severe phenotype.

### 3.8. Immunological Component of Retinal Dystrophies

Retinal dystrophies are believed to have an immunological component, most likely due to the reaction of the immune system to the decaying retinal tissue [45]. The acute onset in Patient 1 effectively mimicked an autoimmune retinopathy and interestingly, the patient reported some improvement on anti-inflammatory treatment. Nevertheless, this warrants further studies and the often serious side effects of immunosuppressive therapy for this inevitably progressive disorder must be weighed.

## 4. Materials and Methods

### 4.1. Evaluation of Slovenian DRAM2 Patients

The study included two female patients with retinal dystrophy in whom genetic analysis identified biallelic *DRAM2* variants, ascertained from Eye Hospital, University Medical Centre Ljubljana, Slovenia. The patients were from two unrelated families, aged 37 (patient 1) and 62 years (patient 2). All investigations were carried out in accordance with the Helsinki Declaration on Biomedical Research in Human Beings. Informed written consent was obtained from the patients.

#### 4.1.1. Genetic and Bioinformatic Analysis

Genetic analysis included whole genome sequencing in Patient 1 and whole exome sequencing in Patient 2. Genomic DNA was extracted from blood samples according to the standard procedure. Sequencing of the defined clinical target was performed using next-generation sequencing in the isolated DNA sample. Briefly, the fragmentation and enrichment of the isolated DNA sample were performed according to the Illumina Nextera Coding Exome capture protocol, with subsequent sequencing on Illumina NextSeq 550 in 2 × 100 cycles (Illumnia, San Diego, CA, USA). After duplicates were removed, the reads were aligned to the UCSC hg19 reference assembly using the Burrows–Wheeler aligner algorithm (BWA) (v0.6.3), and variant calling was performed using a GATK framework (v2.8). Only variants exceeding the quality score of 30.0 and depth of 5 were used for downstream analyses. Variant annotation was performed using the ANNOVAR and snpEff algorithms, with pathogenicity predictions in the dbNSFPv2 database. Reference gene models and transcript sequences are based on the RefSeq database. Structural variants were assessed using the CONIFER v0.2.2 algorithm. Variants with population frequency exceeding 1% in gnomAD, synonymous variants, intronic variants and variants outside the clinical target were filtered out during analyses. An in-house pipeline was used for the bioinformatic analyses of exome sequencing data, in accordance with the GATK best practice recommendations [46].The interpretation of sequence variants was based on ACMG/AMP standards and guidelines [18]. When sequencing the DNA sample, we reached a median coverage of 67× and covered over 99.9% targeted regions with a minimum 10× depth of coverage [47]. The presence of the pathogenic variant in the population was evaluated in the gnomAD database (gnomad.broadinstitute.org, accessed on 31 May 2022). Genetic characteristics of patients were classified into three types based on variant effect predictor (VEP) (https://gnomad.broadinstitute.org/gene/ENSG00000156171?dataset=gnomad_r2_1 (accessed on 31 May 2022)). 

#### 4.1.2. Clinical Examination

Patients underwent a complete ophthalmological exam, including BCVA (Snellen), color vision (Ishihara plates), slit lamp biomicroscopy and dilated fundus examination. Visual field testing was performed using manual kinetic Goldmann perimetry and Octopus G2-top static perimetry. Imaging included color fundus photography with conventional fundus color photographs (Topcon, Tokyo, Japan), FAF and OCT (Spectralis, Heidelberg Engineering, Dossenheim, Germany). The diameter of the hypoautofluorescence lesion (lesion area with a level of darkness of almost 100% in reference to the optic nerve) was measured using ImageJ (U.S. National Institutes of Health, Bethesda, MD, USA). The scale was set using the known approximate 15° distance between the center of the optic nerve and the fovea. ERG was performed according to the standards and guidelines of the International Society for Clinical Electrophysiology of Vision (ISCEV) [48,49,50], using Espion (Diagnosys LLC, Lowell, MA, USA) or RETI scan (Roland Consult Stasche & Finger GmbH, Germany) visual electrophysiology testing systems with HK-loop active electrodes. ffERG was used to assess the general retinal function with the following recording protocols: dark-adapted 0.01 ERG (DA 0.01 ERG; rod-specific response, driven by on-bipolar cells), dark-adapted 3 ERG (DA 3 ERG; DA 3 ERG; combined responses from photoreceptors and bipolar cells, mostly rod-specific), dark-adapted oscillatory potentials (DA osc. pot.; responses mostly from amacrine cells) light-adapted 3 ERG (LA 3 ERG; cone-specific response; a-waves originates from cone photoreceptors and cone off-bipolar cells, while the b-wave arises from on- and off-cone bipolar cells), light-adapted 30 Hz flicker ERG (LA 30 Hz; cone-specific response) [48]. MfERG; patient 1 and 2) [49] and PERG (Patient 1) [50] were used to assess the function of the macula. mfERG testing was performed with the stimuli of 60° in the diameter, presented on a cathode-ray tube monitor. The stimulus included an array of 61 hexagons, which were modulated between light (L) and dark (D) with 96–98% contrast according to a binary m-sequence (511 samples of the sequence: LDDDD). PERG was elicited with 0.8° checkerboard pattern, presented on a 21.6 × 27.8° CRT screen simulator. The checkerboard pattern reversed 1.8 times per second, and the contrast between the black and white fields was 99%. The signals were amplified and stored on a hard disc on the computer for further analysis. Head MRI, chest X-ray and PET-CT were performed in Patient 1. Blood screening in Patient 1 included paraneoplastic panel, tumor markers, rheumatology screening and the exclusion of common infectious ethology.

### 4.2. Review of Previously Published Cases with Variant in DRAM2

The electronic database PubMed was queried (on 20 March 2022) to review previously published cases with retinopathy caused by a pathogenic variant in *DRAM2*. There were no publication year or language restrictions. Inclusion criteria consisted of biallelic variants in *DRAM2* and a description of the phenotype. When FAF images were available, the diameter of the hypoautofluorescence lesion was measured as stated above (Chapter 4.1.2.)

### 4.3. Genotype–Phenotype Analysis

For the purpose of distinguishing whether any genotype results in a milder phenotype, we used the relatively large group of patients with the same, presumably null, genotype (homozygous p.Gly47Valfs*3; N = 11) as the baseline cohort. The p.Ala210GlufsTer16, present in homozygous state in Patient 22, was not considered null, as the variant occurs later on in the gene, potentially escaping the NMD [15], which could result in residual protein function. Similarly, p.1Met? (Patient 1) possibly results in a start codon later on and was therefore also not considered null for this analysis. Patient 26 (p.Leu246Pro homozygous) had no clinical data. 

## 5. Conclusions

In conclusion, a novel start loss variant in the *DRAM2* (p.1Met?) was identified in two Slovenian patients, causing severe RCD in homozygous state and a mild, late-onset macular dystrophy *in trans* with p.Leu246Pro. On the review of all published DRAM2 cases, patients usually present with central visual loss in the third decade and macular abnormalities on fundus examination. OCT findings most commonly reveal outer retinal layer loss, whereas FAF usually shows hyperautofluorescent ring that enlarges towards the periphery during the progression of the disease. ERG findings are most commonly in the pattern of CRD, although macular dystrophy and atypical rod-cone pattern with early macular involvement are also possible. Certain non-null variants such as p.Leu246Pro may result in milder disease.

## Figures and Tables

**Table 1:** The genetic and clinical characteristics of our patients with the novel variant in *DRAM2*.

Patient ID	Genotype	Age at Onset (Years)	Presentation	Age at the First and Last Exam (Years)	BCVA (Snellen Decimal)	Slit Lamp Findings	OCT Characteristics	FAF Characteristics	Visual Field (Octopus G2 Top, MS)	ERG Characteristics
					RE; LE					
Patient 1	c.3G > A (p.Met1?) VUS	35	1 month of relatively rapid deterioration of vision, pericentral visual loss, photopsia	35; 37	1.0; 1.0 0.6; 0.5	1+ to 2+ cells in the vitreous fluid, absent foveal reflex, granular appearance of the macula, parafoveal yellow dots	Loss of photoreceptor layers in the parafoveal region, relative foveal sparing Loss of photoreceptors (ISe) in the foveal and perifoveal regions	Poorly demarcated hypoAF and hyperAF lesions in the macula More widespread hypoAF and hyperAF lesions	Loss of central sensitivity; MS: RE 25 dB, LE 22 dB Worse on follow-up; MS: RE 17 dB, LE 16 dB	Abnormal mfERG and PERG; reduced rod-specific response, delayed cone-specific response on ffERG (cone–rod dystrophy); Worsening: reduced responses on mfERG; undetectable rod-specific responses and severely delayed cone-specific responses on ffERG
Patient 2	c.3G > A (p.Met1?) VUS c.737T > C (p.Leu246Pro) VUS	59	2-year worsening of vision, photophobia	61; 63	0.2; 0.2 0.2; 0.2	Absent foveal reflex, narrow blood vessels, granular appearance of the macula, yellow dots around the vascular arcades	Absent photoreceptors in the fovea, pigment clumping	Central hypoAF and hyperAF ring	Loss of central sensitivity; MS: RE 27 dB, LE 26 dB worse on follow up, MS: BE 24 dB	Abnormal mfERG; ffERG normal (macular dystrophy)

Abbreviations: VUS—variant of unknown significance; BCVA—best corrected visual acuity; RE—right eye; LE—left eye; OCT—optical coherence tomography; FAF—fundus autofluorescence; ERG—electroretinography; mfERG—multifocal ERG; PERG—pattern ERG; ffERG—full-ffield ERG; MS—mean sensitivity; ISe—inner segment ellipsoid. Fundus, OCT and FAF characteristics, visual field and ERG results were highly symmetrical between the eyes. Exam findings from the first and last exam are stated separately in case of differences. Variant calling was based on the GRCh37/hg19 genome assembly.

**Table 2:** Pathogenic variants in DRAM2 associated with retinopathy from our study (Patient 1 and Patient 2) and previously published cases [12,13,14,15,16].

Amino Acid Change	Nucleotide Change	Exon	VEP Annotation	ACMG Classification	Null Variant	gnomAD Allele Frequency	Patient (Reference)
p.Met1?	c.3G > A	3	Start loss	VUS	No	0.00006369	1, 2 (this report)
p.Trp3del	c.8_10delGGT	3	In-frame deletion	Likely pathogenic	No	0.0001240	3 [16]
p.Val16Ala	c.47T > C	3	Missense	VUS	No	0.000003977	4 [14]
p.Ala22del	c.64_66del	3	In-frame deletion	Likely pathogenic	No	0	5 [12,13]
p.Tyr27His	c.79T > C	3	Missense	VUS	No	0	6 [12,13]
p.Ser44Asn	c.131G > A	3	Missense	Likely pathogenic	No	0.00003186	7 [12,13]
p.Gly47Valfs*3	c.140delG	4	Nonsense	Pathogenic	Yes	0.00001196	8–18 [12,13]
p.Gly57Arg	c.169G > C	4	Missense	VUS	No	0	19 [12,13]
p.Val73_Tyr75del	c.217_225del	5	In-frame deletion	Likely pathogenic	No	0	6 [12,13]
p.Arg74His	c.221G > A	5	Missense	VUS	No	0.00001066	20 [16]
p.Gly95Val	c.284G > T	5	Missense	VUS	No	0.00002788	4 [14]
p.His121Leu	c.362A > T	6	Missense	VUS	No	0	21 [12,13]
p.Trp165*	c.494G > A	6	Nonsense	Pathogenic	Yes	0.00003637	7 [12,13]
	c.518-1G > A	7	Splicing	Likely pathogenic	No	0.000004001	24 [15]
p.Ala210Glufs*16	c.628_629insAG	8	In-frame insertion	Likely pathogenic	No	0	22 [15]
p.Tyr226Serfs*2	c.693 + 2T > A c.677dupA	8 8	Splicing Nonsense	Likely pathogenic Likely pathogenic	No Yes	0 0.00002472	25 [15] 26 [17]
p.Arg236_Val237insGly	c.707_709dup	9	In-frame insertion	Likely pathogenic	No	0	23 [16]
p.Leu246Pro	c.737T > C	9	Missense	VUS	No	0.0003508	2 (this report), 26 [17]

Abbreviations: VEP—variant effect predictor; ACMG—American College of Medical Genetics and Genomics; gnomAD—Genome Aggregation Database; VUS—variant of unknown significance.

**Table 3:** The clinical characteristics of previously published cases with variant in *DRAM2*.

Patient ID	Ref.	Gender	Age at Onset (Years)	Symptoms at Onset	Age at the First and Last Exam (Years)	BCVA (Snellen Decimal)		Fundus Characteristics	OCT Characteristics	FAF Characteristics	ERG Characteristics
						RE	LE				
Patient 3 (Kinki-69-1159) p.Trp3del (hom)	[16]	F	Several years before first presentation	Difficulty seeing in dark environments	42; 71	0.05 (42) 0.01 (55) H.M. (71)	0.1 (42) H.M. (55) H.M. (71)	Yellow dots in the macula, color changes of the RPE (42), sparse pigmentation of the retina (71).	Outer retinal layer loss (66).	N/A	ffERG: rod ERG almost nonrecordable, cone ERG reduced (66)
Patient 4 (#119) p.Val16Ala; p.Gly95Val	[14]	N/A	Third decade	Dark adaption difficulties	53	0.6 (53)	0.03 (53)	Granular macular appearance (53).	N/A	Central thinning of photoreceptor layer, paracentral hyperAF ring (53).	ffERG: reduced dark-adapted and light-adapted responses (53)
Patient 5 (BL1) p.Ala22del (hom)	[12,13]	F	30	Central visual loss	40; 44	0.1 (44)	0.1 (44)	Granular macular atrophy, fine yellow dots (44).	Outer retinal layer loss (44).	Enlarging hyperAF ring around central area of hypoAF (40, 44).	mfERG: Severely attenuated (in keeping with macular dysfunction) (30); ffERG: normal (40, 44)
Patient 6 (gc17004) p.Tyr27His; p.Val73_Tyr75del	[12,13]	F	29	Central visual loss	30; 39	0.05 (39)	0.01 (39)	Macular photoreceptor loss, normal peripheral retina (29). Granular macular atrophy, yellow dots, mid-peripheral bone–spicule pigmentation (39).	Outer retinal layer loss (38).	Faint, enlarging hyperAF ring around central area of hypoAF (30; 38); peripheral and midperipheral irregular AF (38).	PERG: severe macular dysfunction (30); mfERG: additional delay (30); ffERG: normal (30), abnormal (dark-adapted and light-adapted) (39)
Patient 7 (1325) p.Ser44Asn; p.Trp165*	[12,13]	F	29	Central visual loss	29; 47	0.1 (47)	0.1 (47)	Macular photoreceptor loss, normal peripheral retina (29). Maculopathy, mid-peripheral degenerations (35). Central macular atrophy, surrounding granular appearance, mid-peripheral bone-spicule pigmentation (47).	Outer retinal layer loss (47).	Faint hyperAF ring around central area of hypoAF (47).	PERG: undetectable (47); ffERG: undetectable (47)
Patient 8 (ES1; IV:10) p.Gly47Valfs*3 (hom)	[12,13]	M	/	Asymptomatic	19	1 (19)	1 (19)	Within normal limits (19).	Reduced foveal thickness, disruption of the ellipsoid zone perifoveally (19).	N/A	N/A
Patient 9 (ES1; IV:7) p.Gly47Valfs*3 (hom)	[12,13]	M	22	Central visual loss	23	0.6 (23)	0.6 (23)	Irregular foveal reflex (23).	Outer retinal layer loss (23).	N/A	N/A
Patient 10 (ES1; IV:9) p.Gly47Valfs*3 (hom)	[12,13]	F	22	Central visual loss	21; 25	0.1 (25)	0.1 (25)	Granular macular atrophy with yellow dots (25).	Reduced foveal thickness (21). Outer retinal layer loss (25).	HyperAF ring around central area of hypoAF (25).	N/A
Patient 11 (ES1; IV:8) p.Gly47Valfs*3 (hom)	[12,13]	F	21	Central visual loss	24	0.2 (21)	0.2 (21)	Granular macular atrophy with yellow dots (24).	Outer retinal layer loss (24).	N/A	N/A
Patient 12 (ES1; IV:11) p.Gly47Valfs*3 (hom)	[12,13]	F	27	Central visual loss	29	0.1 (29)	0.1 (29)	Granular macular atrophy with yellow dots (29).	Outer retinal layer loss (29).	N/A	N/A
Patient 13 (ES1; IV:6) p.Gly47Valfs*3 (hom)	[12,13]	F	26	Central visual loss	32	0.2 (32)	0.2 (32)	Granular macular atrophy with yellow dots, more widespread pigment clumping and irregular reflex extending beyond the macula (32).	Outer retinal layer loss (26).	N/A	N/A
Patient 14 (ES1; III:4) p.Gly47Valfs*3 (hom)	[12,13]	M	23	Central visual loss	56	0.01 (56)	0.01 (56)	Central granular macular atrophy, yellow dots, pigment clumping and mid-peripheral bone–spicule pigmentation (56).	N/A	N/A	N/A
Patient 15 (ES1; III:13) p.Gly47Valfs*3 (hom)	[12,13]	M	25	Central visual loss	46	0.01 (46)	0.01 (46)	Central macular atrophy, pigment clumping, surrounding granular appearance, and mid-peripheral bone–spicule pigmentation (46).	N/A	N/A	N/A
Patient 16 (ES1; III:5) p.Gly47Valfs*3 (hom)	[12,13]	F	28	Central visual loss	51	0.1 (51)	0.1 (51)	Central macular atrophy, surrounding granular appearance, pigment clumping in LE and minimal peripheral changes (51).	N/A	N/A	N/A
Patient 17 (ES1; III:6) p.Gly47Valfs*3 (hom)	[12,13]	M	25	Central visual loss	37	0.05 (37)	0.05 (37)	Granular macular atrophy with yellow dots (37).	Outer retinal layer loss (37).	N/A	PERG: severely subnormal (37); ffERG: normal (37)
Patient 18 (ES; III:1) p.Gly47Valfs*3 (hom)	[12,13]	M	16	Central visual loss	48	0.005 (48)	0.005 (48)	Central macular atrophy, pigment clumping, surrounding granular appearance and heavy mid-peripheral bone–spicule pigmentation (48).	N/A	N/A	N/A
Patient 19 (PCI1) p.Gly57Arg (hom)	[12,13]	F	35	Central visual loss	43	0.3 (43)	0.4 (43)	Central macular atrophy, surrounding granular appearance (43).	Outer retinal layer loss (43).	HyperAF ring around central area of hypoAF (43).	ffERG: normal (normal dark-adapted, borderline light-adapted responses) (42)
Patient 20 (Kinki-12-1022) p.Arg74His (hom)	[16]	M	36	Reduction of visual acuity.	42; 71	0.2 (36) 0.03 (55) H.M. (71)	0.09 (36) 0.03 (55) H.M. (71)	Macular and mid-peripheral RPE degeneration (42). Diffuse retinal degeneration, bone–spicule pigmentation (71).	Outer retinal layer loss (71).	Mosaic pattern with hyperAF and hypoAF (71).	ffERG: dark-adapted ERG nonrecordable, light-adapted ERG reduced (51)
Patient 21 (gc4728) p.His121Leu (hom)	[12,13]	M	34	Central visual loss	34; 47	0.05 (47)	0.05 (47)	Macular photoreceptor loss, normal peripheral retina (34). Central macular atrophy, pigment clumping, yellow dots, mid-peripheral bone–spicule pigmentation (47).	Outer retinal layer loss (44).	Faint, enlarging hyperAF ring around central area of hypoAF (37;45); midperipheral irregular AF (45).	PERG: severe macular dysfunction (34); mfERG: additional delay (47); ffERG: normal (34) abnormal (dark- and light-adapted responses) (47)
Patient 22 (Fi19/01; I) p.Ala210GlufsTer16 (hom)	[15]	M	45	Progressive visual loss, photophobia	47; 52	0.6 (47) 0.05 (52)	0.5 (47) 0.05 (52)	Macular RPE disturbances (47). Macular RPE atrophy, mild mid-peripheral disturbances (52).	Foveal thinning due to severe atrophy of the photoreceptor and RPE layers (47).	Perifoveal hypoAF granularity (47). Increasing hypoAF with a more marked perifoveal pattern (50).	ffERG: very low responses under dark- and light-adapted conditions (52)
Patient 23 (Jikei-176-1241) p.Arg236_Val237insGly (hom)	[16]	F	19	Difficulty in night vision (19), reduced visual acuity (37)	38; 43	0.3 (38) 0.2 (43)	0.6 (38) 0.3 (43)	Fine white dots in the macula (38), granular macular degeneration (43).	Disrupted ellipsoid zone and thinning of the outer retinal layers in the macula (38).	Macular hypoAF surrounded by hyperAF (38), hypoAF in the midperiphery (43).	ffERG: almost nonrecordable dark- and light-adapted responses (43)
Patient 24 (Fi19/02; II) c.518-1G > A (hom)	[15]	F	27	Progressive visual loss, photophobia	30; 37	0.2 (30) 0.1 (38)	0.2 (30) 0.1 (38)	RPE disturbances in the macula (which respected the fovea), mild mid-peripheral RPE disturbances, few bone–spicule pigmentations (30). Macular RPE atrophy, disturbances, and bone–spicule pigmentations in the midperiphery (37).	Outer retinal layers loss (30). Complete macular atrophy (especially in the central fovea), increased disturbances in the mid-periphery (37).	Perifoveal hypoAF, mid-peripheral mottled hypoAF (30). Marked macular hypoAF, mid-peripheral disturbances more widespread and severe (37).	N/A
Patient 25 (Fi19/03; III) c.693 + 2T > A (hom)	[15]	F	27	Visual decline, photophobia	52; 57	0.05 (52)	0.1 (52)	Macular atrophy, peripheral RPE disturbances, bone–spicule pigmentations (52). Without significant progression (57).	Outer retinal layers loss (52).	Macular and peripheral hypoAF (52). Diffuse RPE disturbances in mid-periphery and periphery (54).	ffERG: complete abolishment of cone and rod function (52)

Abbreviations: Ref.—reference from the original article; BCVA—best corrected visual acuity; OCT—optical coherence tomography; FAF—fundus autofluorescence; RE—right eye; LE—left eye; ERG—electroretinography; PERG—pattern ERG; mfERG—Multifocal electroretinography; ffERG—full field ERG; hom—homozygous; N/A—not available; H.M.—hand motions; RPE—retinal pigment epithelium; AF—autofluorescent. At Patient ID, the denomination (Patient ID and/or family name) from the original name is given in round brackets. Below the patient ID the reference from the original article is given.

## Data Availability

The original data are available upon reasonable request to the corresponding author.

## Abbreviations

IRD	Inherited retinal dystrophy
RPE	Retinal pigment epithelium
DRAM2	DNA-damage regulated autophagy modulator 2
gnomAD	Genome Aggregation Database
dbSNP	Single Nucleotide Polymorphism Database
ACMG/AMP	American College of Medical Genetics and Genomics and the Association for Molecular Pathology
BE	Both eyes
BCVA	Best corrected visual acuity
FAF	Fundus autofluorescence imaging
OCT	Optical coherence tomography
MS	Mean sensitivity
RE	Right eye
LE	Left eye
ERG	Electroretinography
PERG	Pattern electroretinography
mfERG	Multifocal electroretinography
ffERG	Full-field electroretinography
MRI	Magnetic resonance imaging
PET-CT	Positron emission tomography and computed tomography
ZIC4	Zinc Finger Protein 4
ANA	Antinuclear antibody
MMF	Mycophenolate Mofetil
ISe	Inner segment ellipsoid
CRD	Cone-rod dystrophy
RCD	Rod-cone dystrophy
CD	Cone-dystrophy
RP	Retinitis pigmentosa
PTC	Premature termination codon
NMD	Nonsense mediated decay
UCSC	University of California Santa Cruz
BWA	Burrows-Wheeler aligner
GATK	Genome Analysis Toolkit
CONIFER	Copy Number Inference Form Exome Reads
VEP	Variant Effect Predictor
